# Nuclear oxidative damage correlates with poor survival in colorectal cancer

**DOI:** 10.1038/sj.bjc.6604821

**Published:** 2008-12-09

**Authors:** J Sheridan, L-M Wang, M Tosetto, K Sheahan, J Hyland, D Fennelly, D O'Donoghue, H Mulcahy, J O'Sullivan

**Affiliations:** 1 Centre for Colorectal Disease, St Vincent's University Hospital, Elm Park Dublin 4, Republic of Ireland

**Keywords:** 8-oxo-dG, oxidative damage, colon, mitochondria, survival, apoptosis

## Abstract

Oxidative DNA damage results from DNA adducts such as 8-oxo-7, 8 dihydro-2′-deoxyguanosine (8-oxo-dG), which is a pro-mutagenic lesion. No known association between 8-oxo-dG, disease progression and survival exists in colorectal cancer (CRC). We examined levels of 8-oxo-dG in sporadic CRC to determine its relationship with pathological stage and outcome. A total of 143 CRC patients and 105 non-cancer patients were studied. Nuclear and cytoplasmic 8-oxo-dG was assessed using immunohistochemistry. Double immunofluorescence using 8-oxo-dG and manganese superoxide dismutase (MnSOD) antibodies localised cytoplasmic 8-oxo-dG. Apoptosis was detected using TUNEL. Nuclear staining levels were similar in tumour tissue and matched normal mucosa in both epithelial (*P*=0.22) and stromal (*P*=0.85) cells. Epithelial cytoplasmic staining was greater in tumour tissue (*P*<0.001). Double immunofluorescence localised cytoplasmic 8-oxo-dG to mitochondria. Epithelial and stromal nuclear 8-oxo-dG decreased with local disease spread, but highest levels were found in distant disease (*P*<0.01). Survival was related to epithelial nuclear and stromal staining in normal mucosa (*P*<0.001) and tumour (*P*<0.01) but was unrelated to cytoplasmic staining. Normal control cells in tissue from cancer patients with high levels of 8-oxo-dG failed to undergo cell death. 8-oxo-dG may be an important biomarker of disease risk, progression and survival for CRC patients.

Colorectal cancer is one of the commonest cancers in the Western World (Ferlay *et al*, 2007) and genetic events associated with tumour initiation and progression have been extensively studied (Fearon and Vogelstein, 1990). The majority of mutations found in sporadic colorectal cancer are due to single-base substitutions (Sjoblom *et al*, 2006) such as GC to TA transversions.

Oxidative stress is defined as a disturbance in the balance between the production of reactive oxygen species and antioxidant defences. It is associated with aging and implicated in a variety of disease processes including atherosclerosis, diabetes mellitus and cancer (Pacifici and Davies, 1991; Maritim *et al*, 2003; Valko *et al*, 2004; Minuz *et al*, 2006). Endogenous sources of reactive oxygen species production include oxidative phosphorylation and inflammatory cell activation, and despite multiple conserved redox modulating systems, a proportion of reactive oxygen species continuously escape the mitochondrial respiratory chain. The significance of reactive oxygen species within antioxidant systems and its role in carcinogenesis is not fully understood. Enhanced antioxidant mechanisms in gastrointestinal tumour cells *in vivo* are associated with chemo-resistance, metastasis and poor prognosis, whereas many *in vitro* studies suggest that antioxidant enzymes have tumour-suppressing properties (Janssen *et al*, 1998; Toh *et al*, 2000), although the majority of conflicting results may be explained by reference to the net redox status of malignant cells (Kinnula and Crapo, 2004).

The DNA adduct, 8-oxo-7, 8-dihydro-2′-deoxyguanine (8-oxo-dG) is formed by the reaction of OH radical with the DNA guanine base. It is a pro-mutagenic lesion that mispairs with adenine leading to GC to TA transversions. This oxidative DNA damage is repaired primarily by the base excision repair pathway incorporating the glycosylases OGG1, MYH and NUDT1 (Chow *et al*, 2004). Few data are available on 8-oxo-dG levels in colorectal diseases (Dincer *et al*, 2007) and the levels of 8-oxo-dG in relation to disease progression and survival has not been studied in colorectal tumours. We assessed nuclear and mitochondrial 8-oxo-dG staining in tumour and matched normal tissues and in tissues from a non-cancer control population.

## Materials and methods

### Patients and tissue collection

This study included 143 randomly selected patients with colorectal cancer (median age 67 years; range 29–87 years; 73 men, 70 women) treated surgically in the Centre for Colorectal Disease, St Vincent's University Hospital, Dublin, Ireland between November 1991 and August 2002. We also studied 105 consecutive patients (median age 45 years; range, 17–87 years; 41 men, 64 women) undergoing colonoscopy for investigation of altered bowel habit and whose investigations were normal. These investigations included histological examination of colonic biopsies, haematological indices and biochemical inflammatory markers (erythrocyte sedimentation rate and C reactive protein).

Following surgery, tumours were fixed in 1% formalin and embedded in paraffin. Tumours were staged using the WHO classification (Morson, 1976). There were 13 Stage I, 46 Stage II, 39 Stage III and 45 Stage IV cancers. Colorectal mucosal tissue remote from the tumour and adjacent to histologically normal mucosa was obtained for 99 of the 143 cases. Patients were followed up at 6 monthly intervals and median follow up of patients alive at the end of study was 6.7 years. Ethical approval was granted by the St Vincent's Hospital Ethics and Medical Research Committee to conduct this study.

### Tissue microarray construction

For all cancer cases, haematoxylin- and eosin-stained slides from formalin-fixed, paraffin-embedded colorectal tumour blocks were used to identify specific areas of the cancer. These areas were aligned with the tissue block and four 6 mm cores taken and transferred to a recipient block using a Tissue Microarrayer (Beecher Instruments, Silver Spring, MD, USA). Here, 4 *μ*m sections were cut for immunohistochemical studies and mounted onto SuperFrost Plus adhesive slides (Menzel-Glaser, Braunschweig, Germany).

### 8-oxo-dG immunohistochemistry

Immunohistochemistry was performed using colorectal tissue microarray slides and the DAKO ChemMate Envision Kit (Dako, Glostrup, Denmark). Then, 4 *μ*m sections were baked for 30 min at 90°C, deparaffinised in xylene and re-hydrated in alcohol and deionised water. All slides in this study were processed and stained on the same day.

Antigen retrieval was performed using 1500 ml antigen retrieval solution (15 ml of 1 M sodium citrate and 15 ml of 1 M citric acid in deionised water, pH 6.0). This solution was heated in a pressure cooker (Menarini Diagnostics, Berkshire, England) with the lid off for 20 min at full power in an 800 W microwave. Slides were placed in the pressure cooker, lid sealed and heated at full power until the pressure valve popped up (5–7 min). Slides remained incubated at full power for a further 4 min. The pressure cooker was removed from the microwave and the lid removed once the pressure equalised (takes approximately 10 min). Slides were washed in PBS and 0.05% Tween for 10 min. Non-specific binding was blocked using 10% casein in PBS for 10 min at room temperature. 8-oxo-dG mouse monoclonal antibody (Genox, Baltimore, MD, USA) was diluted 1 : 40 in antibody diluent (Dako) and incubated on slides for 2 h at room temperature in a humidified chamber. An IgG control antibody and the elimination of the primary antibody were used as negative controls.

Endogenous peroxidase activity was blocked using 3% hydrogen peroxide for 7 min at room temperature. Slides were washed as stated above followed by incubation with secondary antibody/HRP (Dako) for 30 min at room temperature.

Slides were washed and incubated with 1 : 50 DAB in substrate buffer for 10 min at room temperature, followed by a PBS wash and placed in running water for 5 min. DAB staining was intensified by application of 0.5% copper sulphate to each slide for 10 min. Slides were washed and counterstained with 1 : 4 Mayer's haematoxylin (BDH Laboratories, Poole, UK) for 20 s followed by a water wash for 5 min.

Tissues were dehydrated and mounted using Pertex mounting media and images were captured using Olympus DP50 light microscope and AnalySIS software (Soft Imaging System Corporation, Lakewood, CO, USA).

### Immunohistochemical scoring

Four cores were examined for each case and both epithelial and stromal cells were assessed for percentage of nuclear and cytoplasmic cells staining positivity and associated nuclear and cytoplasmic staining intensity. Intensity was graded as 0 (negative), 1 (weak), 2 (moderate) and 3 (strong). The median value of each parameter was calculated from the results of the four cores. The scoring was by consensus review (JS and JOS). The reviewers were blinded to the original diagnosis and the patient outcomes at the time of review.

### Immunofluorescence

Immunofluorescence was performed using monoclonal 8-oxo-dG and polyclonal manganese superoxide dismutase (MnSOD) antibodies. Antigen retrieval was performed as described above. Non-specific binding was blocked using 10% casein in PBS for 10 min at room temperature. An antibody mix of 8-oxo-dG mouse monoclonal antibody (Genox) (1 : 40 dilution) and rabbit anti MnSOD polyclonal antibody (Nventa Biopharmaceuticals, San Diego, CA, USA) (1 : 200 dilution) was incubated on slides for 2 h at room temperature in a humidified chamber. Negative controls incorporated neither of the primary antibodies. Biotinylated horse anti-mouse antibody (1 : 200 dilution) (Vector Laboratories, Peterborough, UK) and incubated on slides for 30 min. Mouse and biotin CY3 (SigmaAldrich, Gillingham, UK) (1 : 100 dilution) and goat and rabbit FITC (SigmaAldrich) (1 : 50 dilution) fluorescent secondary antibodies were applied to slides for 30 min. Slides were counterstained with a 10 ng ml DAPI (SigmaAldrich) for 5 min. Slides were incubated with prolonged anti-fade solution and immunofluorescence images were captured using a Zeiss fluorescence microscope.

### TUNEL and 8-oxo-dG immunofluorescence

Apoptotic cells within the tissue were detected by terminal deoxynucleotidyl transferase-mediated dUTP nick-end labelling (TUNEL). Slides were treated with the Fluorometric Apoptosis (Promega, Madison, WI, USA) according to the instructions of the manufacturer for paraffin-embedded tissues. Here, 8-oxo-dG immunofluorescence was performed as described above.

### Statistical analysis

Continuous data are presented as medians and interquartile ranges. Non-parametric data were assessed using Wilcoxon's rank sum test, Wilcoxon's signed rank test, the Kruskal–Wallace test or Spearman's rank correlation coefficient as appropriate.

Differences between proportions were assessed using the *χ*^2^ test. The distribution of 8-oxo-dG staining was divided into tertiles to develop usable groups to determine the effect of staining on survival. The resulting groups contained different numbers of patients, but were as close to tertiles as it was possible to achieve. Kaplan–Meier survival curves were constructed with cancer-related mortality as the end point. Differences in survival between groups were assessed using the log-rank test. Multivariate survival analyses were performed with the Cox proportional hazards model using the Statistical Package for the Social Sciences (SPSS, Chicago, IL, USA). All *P*-values are two-sided and *P*-values less than 0.05 were considered statistically significant in all analyses.

## Results

### 8-oxo-dG staining in tumour *vs* matching normal non-adjacent mucosa in cancer patients

**Figure 1:**
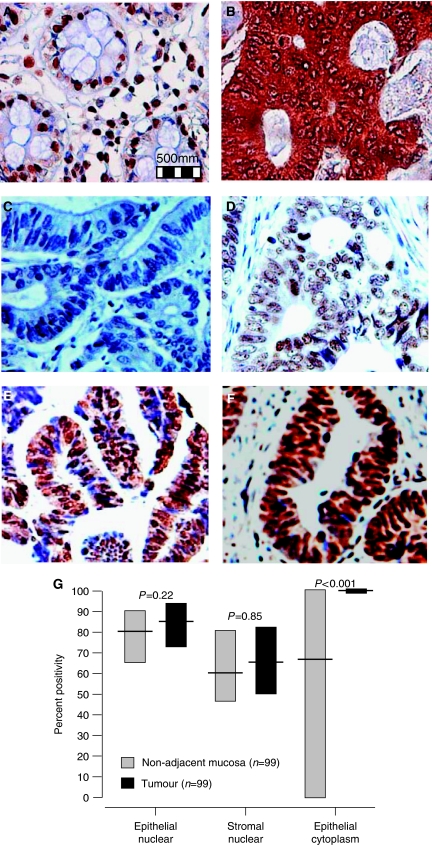
(**A**) 8-oxo-dG nuclear staining (weak cytoplasmic staining) in normal non-adjacent mucosa *vs* (**B**) strong 8-oxo-dG cytoplasmic staining in the matching tumour tissue. (**C**–**F**) Represent negative, weak, moderate and strong intensity levels in tumour epithelial cells. (**G**) Graphical representation of 8-oxo-dG percent positivity in normal non-adjacent mucosa *vs* matched tumour tissue in cancer patients using Wilcoxon's signed rank test.

Figure 1A and B shows representative images of 8-oxo-dG staining in normal adjacent mucosa from a cancer patient and matching tumour tissue respectively. High levels of nuclear staining is evident in normal non-adjacent epithelial cells (Figure 1A), whereas high levels of cytoplasmic staining is evident in tumour epithelial cells (Figure 1B). Panels c–f show representative images of the different nuclear 8-oxo-dG intensities ranging from negative to weak, moderate and strong.

Figure 1G illustrates graphically the percentage of positive cells and cellular localisation of 8-oxo-dG in tumour when compared with non-adjacent normal tissue. Percentage nuclear staining was similar in matched normal non-adjacent mucosa and tumour tissue in both epithelial (*P*=0.22) and stromal (*P*=0.85) cells. However, percentage epithelial cytoplasmic staining was greater in tumour tissue (*P*<0.001) 8- oxo-dG staining in both tumour tissues and non-adjacent mucosa was unrelated to age (data not shown).

### Immunofluorescence co-localisation of MnSOD and 8-oxo-dG in tumour

**Figure 2:**
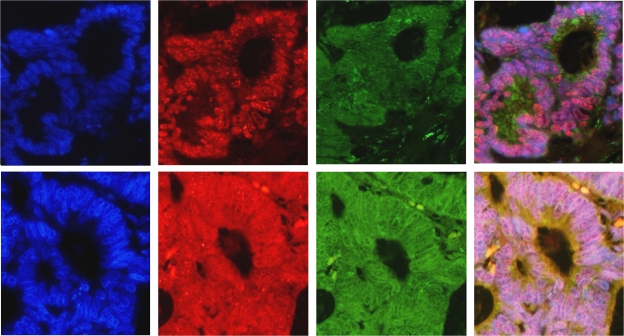
Immunofluorescence co-localisation of 8-oxo-dG and MnSOD. DAPI DNA stain (blue), 8-oxo-dG (red) and MnSOD (green). Upper panels, a tumour case with no cytoplasmic 8-oxo-dG (shows nuclear 8-oxo-dG) with abundant cytoplasmic MnSOD staining. Lower panels, represent a tumour case with abundant cytoplasmic 8-oxo-dG and MnSOD demonstrating co-localisation (yellow). Magnification × 40.

Double immunofluorescence staining of 8-oxo-dG and MnSOD showed that cytoplasmic 8-oxo-dG is mitochondrial in nature. Figure 2 (upper panel) illustrates a colorectal cancer case where no 8-oxo-dG cytoplasmic staining is detected (no red staining) but abundant cytoplasmic MnSOD staining is evident (green). In contrast, Figure 2 (lower panel) shows co-localisation of cytoplasmic 8-oxo-dG and MnSOD staining (yellow).

### 8-oxo-dG levels and tumour progression

**Figure 3:**
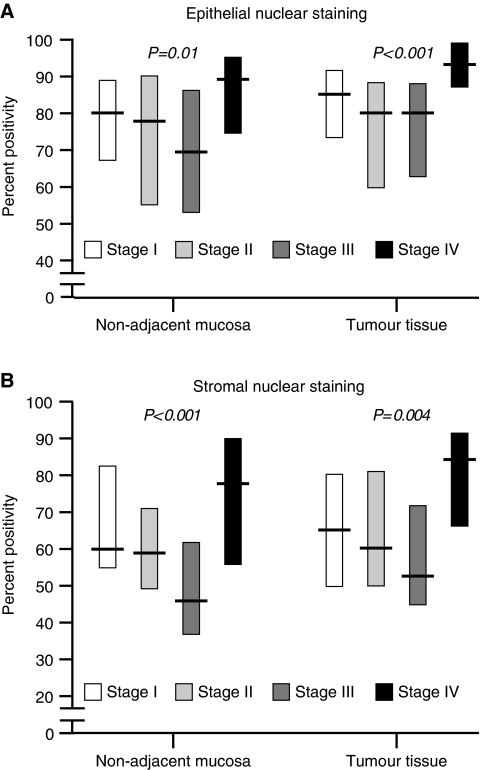
Graphical representation of 8-oxo-dG nuclear percent positivity in epithelium (**A**) and stromal cells (**B**) for each colorectal cancer disease stage.

**Figure 4:**
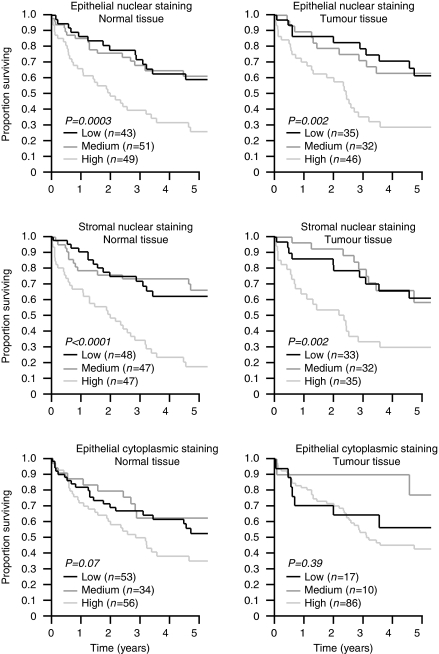
Survival of colorectal cancer patients stratified by epithelial and stromal 8-oxo-dG staining.

Figure 3A and B shows the relationship between epithelial and stromal nuclear 8-oxo-dG staining and anatomical progression. Levels were relatively high in early stage disease and tended to decrease with advancing local spread. However, highest levels were found in the primary tumours of patients with distant metastases (stage IV) and similar patterns were seen in the epithelial and stromal nuclei of both tumour and non-adjacent normal mucosal cells. Cytoplasmic staining was unrelated to stage (data not shown). Long-term survival was closely associated with tumour stage (*P*<0.0001) (data not shown). In addition, poor outcome was related to high levels of 8-oxo-dG epithelial nuclear staining in normal non-adjacent mucosa (uncorrected log-rank test, *P*=0.0003 (corrected for age, gender and tumour stage in the proportional hazards model, relative risk 2.2; 95% confidence intervals 1.3–3.6, *P*=0.002)) and tumour tissue (uncorrected, *P*=0.002 (corrected, RR 1.7; 95% CI 0.8–3.4; *P*=0.15)). Poor outcome was also associated with high levels of stromal nuclear staining in normal non-adjacent mucosa (uncorrected, *P*<0.0001 (corrected, RR 2.2; 95% CI 1.3–3.8; *P*=0.005)) and tumour tissue (uncorrected, *P*=0.002 (corrected, RR 2.7; 95% CI 1.3–5.5; *P*=0.006)). Survival was unrelated to cytoplasmic staining in either tumour or normal mucosa (Figure 4). Staining intensity levels did not correlate with survival (data shown).

Secondary analyses were performed to include only those patients undergoing curative surgery. Again, survival, corrected for age, gender and tumour stage was related to 8-oxo-dG epithelial nuclear staining in normal non-adjacent mucosa (RR 3.4; 95% CI 1.6–7.4; *P*=0.002) and tumour tissue (RR 4.1; 95% CI 1.3–12.5; *P*=0.01), and with stromal nuclear staining in normal non-adjacent mucosa (RR 3.5; 95% CI 1.5–8.1; *P*=0.003) but not with stromal nuclear staining in tumour tissue (RR 2.5; 95% CI 0.7–8.7; *P*=0.14).

### 8-oxo-dG staining in normal controls

Epithelial nuclear and cytoplasmic and stromal nuclear 8-oxo-dG staining was also examined in the colonic mucosa of 105 patients without structural bowel diseases to determine the effect of age and gender on 8-oxo-dG staining. No significant associations were found (data not shown).

### 8-oxo-dG levels and apoptosis in cancer patients and normal controls

**Figure 5:**
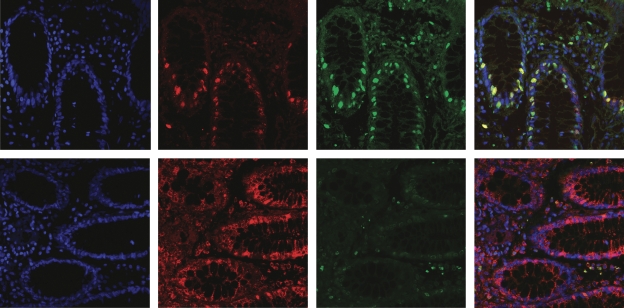
Representative confocal microscopy images of dual labelling TUNEL (green fluorescence) and 8-oxo-dG (red fluorescence). Upper panels – normal colonic cells from patients without colorectal cancer that have high levels of 8-oxo-dG are seen to undergo apoptosis. Lower panels – normal colonic cells from cancer patients with high levels of 8-oxo-dG fail to undergo apoptosis.

Representative confocal microscopy images of dual labelling TUNEL (green fluorescence) and 8-oxo-dG (red fluorescence) are shown in Figure 5. Normal colonic cells in tissue from patients without colorectal cancer who have high levels of 8-oxo-dG are seen to undergo apoptosis (upper panels). However, normal colonic cells in tissue from cancer patients with high 8-oxo-dG levels fail to undergo apoptosis (lower panels).

## Discussion

Several methods have been developed to measure 8-oxo-dG levels in biologic samples. These include high performance liquid chromatography coupled with electrochemical detection, gas chromatography and ^32^P postlabelling (Halliwell, 1999). However, results from these labour-intensive methods have been difficult to reproduce and there is disagreement both with regard to actual values and variability within cells and tissue specimens. Indeed, a European Standards Committee on Oxidative DNA Damage (ESCODD) has been established because of such discrepancies with identification of drawbacks associated with these methods (Gedik and Collins, 2005). In this study, we used immunohistochemical methods as it allowed us to localise 8-oxo-dG to different cell types without the risk of artifactual production of 8-oxo-dG associated with DNA extraction and hydrolytic processes. This antibody is specific to oxidised DNA and does not recognise RNA (Toyokuni *et al*, 1997).

We found substantial staining of 8-oxo-dG in both the epithelial and stromal nuclei of tissues from the normal colon of both control and cancer patients in addition to cancer tissues taken from the latter. High levels of 8-oxo-dG have previously been detected in the blood of patients with various cancers including oesophagus, lung and colon (Gackowski *et al*, 2002, 2005; Breton *et al*, 2005) whereas oxidative stress is also linked to chronic inflammatory states including inflammatory bowel diseases, known to be associated with colorectal cancer risk (Pohl *et al*, 2000; Dincer *et al*, 2007). Other risk factors for colorectal cancer include obesity and insulin resistance, themselves closely associated with oxidative stress (Urakawa *et al*, 2003) and it is clear that these various interrelationships are complex. Mechanisms whereby oxidative damage could result in cancer development have been proposed and a system that includes oxidative damage and stress resulting in the formation of pro-mutagenic 8-oxo-dG with subsequent widespread G:C to T:A transversions supports the mutator phenotype hypothesis (Loeb *et al*, 1974). Such a hypothesis seems plausible, although still remaining speculative (Olinski *et al*, 2003).

We studied 8-oxo-dG staining in the colonic tissues of a large number of patients without structural bowel disease and found no association between oxidative damage and age. In relation to our cancer cohort, we found that tumour tissues had higher levels of cytoplasmic 8-oxo-dG than non-adjacent colonic mucosa. However, the main finding from our study was that high levels of 8-oxo-dG were associated with poor survival, both in uncorrected analyses and when corrected for tumour stage. This remained true even when patients with metastatic disease were excluded in secondary analyses. It is also clear from a review of Figure 4 that it was only those patients with the highest levels of nuclear 8-oxo-dG staining who were at increased risk and that those with medium staining levels behaved similarly to those with low staining levels.

Our cutoff points were constructed arbitrarily by dividing our cohort into tertiles, but it is probable that much more powerful associations with outcome would be found using additional discriminatory analyses such as the maximal log-rank test (Peto *et al*, 1977). Our findings relating to colorectal cancer have not been described earlier, but high levels of 8-oxo-dG have been associated with poor outcome in non small cell lung cancer and renal cell carcinoma (Miyake *et al*, 2004; Shen *et al*, 2007) whereas oxidative damage in non-cancerous liver tissues also appears to predict recurrence of hepatocellular cancer (Matsumoto *et al*, 2003).

As survival was associated with 8-oxo-dG staining and also closely associated with anatomical spread, one might have expected a positive association between 8-oxo-dG staining and advancing tumour stage. However, this was not the case, especially in those undergoing curative surgery. If anything, 8-oxo-dG nuclear staining was relatively high in early disease and declined somewhat with advancing tumour stage until the development of distant metastases when there was a sharp rise again. Thus, the effect of intranuclear oxidative damage on clinical progression was clearly independent of pathological stage, especially in those without distant metastases, as confirmed by our multivariate analyses. In contrast, although we found considerable cytoplasmic staining in epithelial cells, and further identified it as mitochondrial in nature, it is clear that this widespread mitochondrial damage was unimportant with regard to both anatomical and clinical disease progression.

What is the pathophysiological explanation for our findings in patients with and without colorectal cancer? Previous studies suggest that 8-oxo-dG accumulation may be due to decreased DNA repair capacity (Gackowski *et al*, 2003). We found that colonic cells with high levels of nuclear 8-oxo-dG taken from non-colorectal cancer patients readily undergo apoptosis. However, in cancer patients, normal colonic cells with high levels of 8- oxo-dG did not undergo cell death and it is possible that the mutator phenotype (Loeb *et al*, 2003) may predispose these cells to further accumulation of mutations and genomic instability. As to our findings in the different colorectal cancer stages, it is possible that the high levels of nuclear oxidative damage found in early stage disease may contribute to this mutator phenotype with a subsequent decline as tumours progress locally. In those with distant metastases, the sharp rise in nuclear 8-oxo-dG levels may correlate with a more hypoxic tissue environment in late stage metastatic disease with upregulation of HIF-1 and VEGF levels (Jiang *et al*, 2003) and it is clear that rises also relate to clinical progression independent of tumour stage. Ultimately, the role of 8-oxo-dG and reactive oxygen species in colorectal cancer remains speculative, but numerous epidemiological, *in vitro* and *in vivo* animal studies suggest that oxidative damage may be important in cancer initiation and progression and that antioxidants may be cytoprotective. It will be interesting to further assess the precise contribution made by 8-oxo-dG and similar compounds to these systems.
